# PbS Quantum Dots-Decorated BiVO_4_ Photoanodes for Highly Efficient Photoelectrochemical Hydrogen Production

**DOI:** 10.3390/nano13050799

**Published:** 2023-02-22

**Authors:** Joo-Won Seo, Seung-Beom Ha, In-Cheul Song, Jae-Yup Kim

**Affiliations:** Department of Chemical Engineering, Dankook University, Yongin 16890, Republic of Korea

**Keywords:** photoelectrochemical, hydrogen production, PbS, quantum dots, BiVO_4_

## Abstract

While metal oxides such as TiO_2_, Fe_2_O_3_, WO_3_, and BiVO_4_ have been previously studied for their potential as photoanodes in photoelectrochemical (PEC) hydrogen production, their relatively wide band-gap limits their photocurrent, making them unsuitable for the efficient utilization of incident visible light. To overcome this limitation, we propose a new approach for highly efficient PEC hydrogen production based on a novel photoanode composed of BiVO_4_/PbS quantum dots (QDs). Crystallized monoclinic BiVO_4_ films were prepared via a typical electrodeposition process, followed by the deposition of PbS QDs using a successive ionic layer adsorption and reaction (SILAR) method to form a p-n heterojunction. This is the first time that narrow band-gap QDs were applied to sensitize a BiVO_4_ photoelectrode. The PbS QDs were uniformly coated on the surface of nanoporous BiVO_4_, and their optical band-gap was reduced by increasing the number of SILAR cycles. However, this did not affect the crystal structure and optical properties of the BiVO_4_. By decorating the surface of BiVO_4_ with PbS QDs, the photocurrent was increased from 2.92 to 4.88 mA/cm^2^ (at 1.23 V_RHE_) for PEC hydrogen production, resulting from the enhanced light-harvesting capability arising from the narrow band-gap of the PbS QDs. Moreover, the introduction of a ZnS overlayer on the BiVO_4_/PbS QDs further improved the photocurrent to 5.19 mA/cm^2^, attributed to the reduction in interfacial charge recombination.

## 1. Introduction

Growing concern over air pollution and global warming caused by the extensive burning of fossil fuels has led to an increased focus on producing and utilizing carbon-neutral energy sources [1,2,3,4,5,6,7,8,9,10,11,12,13,14,15]. One promising approach for the generation of clean and renewable energy is photoelectrochemical (PEC) hydrogen production. In a typical PEC device, either a photoanode or a photocathode serves as the working electrode for light harvesting and either the oxygen evolution reaction (in the case of the photoanode) or the hydrogen evolution reaction (in the case of the photocathode) while the other half-reaction takes place at the counter electrode [16]. Under illumination, for example, the photoanode absorbs photons with energies greater than its band-gap energy, generating electron–hole pairs. The photoelectrons are then transported and collected to the conducting substrate through the conduction band (CB) of the photoanode, while the remaining holes participate in the oxidation reaction. The collected photoelectrons then flow to the counter electrode through an external circuit where they participate in the hydrogen evolution reaction. To achieve efficient PEC hydrogen production, the photoanode must have a suitable band-gap for broad light absorption, superior charge transport properties, and good photo- and chemical stabilities.

To this end, various metal oxides, such as TiO_2_ [5,6], Fe_2_O_3_ [7,8], ZnO [9,10], and BiVO_4_ [11,12,13,14,15], have been intensively studied for use in PEC photoelectrodes due to their intrinsic chemical stability in water and low materials cost. BiVO_4_, in particular, has garnered significant attention owing to its excellent chemical stability, low toxicity, and relatively narrow band-gap of 2.4 eV. However, the photocurrent density of the BiVO_4_ photoanode reported thus far falls short of the theoretical maximum (~7.5 mA/cm^2^), primarily due to severe charge recombination and slow surface catalytic kinetics [12]. Furthermore, while the band-gap of BiVO_4_ is relatively small compared to other metal oxides, it is not sufficient to cover the entire visible range.

To enhance the PEC properties of BiVO_4_, particularly its light-harvesting capability, combining it with narrow band-gap quantum dots (QDs) may offer a promising solution. QDs possess unique optoelectronic properties that arise from the quantum confinement effect, including a high extinction coefficient, band-gap tunability, and potential for multiple exciton generation [17,18,19]. While several types of QDs, such as carbon QDs, graphene QDs, and CeO_2_ QDs have been investigated for use in combination with BiVO_4_ to improve its light-harvesting capability [13,20,21,22], there has been a lack of sufficient research on the use of typical narrow band-gap QDs, such as PbS, PbSe, and Cu-In-Se QDs, for sensitizing the BiVO_4_ photoelectrode.

In this study, we present a highly efficient photoanode for PEC hydrogen production based on a p-n heterojunction of BiVO_4_/PbS QDs using a simple and facile process. BiVO_4_ films were prepared by a typical electrodeposition process, followed by the decoration of PbS QDs on the surface using a successive ionic layer adsorption and reaction (SILAR) method. This is the first time that narrow band-gap QDs were applied to sensitize a BiVO_4_ photoelectrode. While various binary metal chalcogenides (e.g., PbS, CdS, CdSe, ZnS, ZnSe, MoS_2_, Bi_2_S_3_, In_2_S_3_, Cu_2_S, NiS, etc.) and ternary metal chalcogenides (e.g., CuInS_2_, CuInSe_2_, CuGaS_2_, etc.) have been studied for their potential use in PEC and photocatalytic hydrogen evolution [23,24,25], we selected the PbS QDs for their ease of deposition using a simple SILAR process and their impressive PEC performance reported in previous work [26]. We characterized the nanostructures and chemical states of the photoelectrodes and examined the influence of PbS QD sensitization on the PEC hydrogen production performances. Additionally, ZnS overlayers were coated on the surface of the BiVO_4_/PbS QDs using SILAR to reduce charge recombination at the QD-based photoelectrode surface, which is a commonly used method [27,28]. The improved light-harvesting capability resulting from the narrow band-gap of PbS and enhanced charge transfer properties led to a significantly higher photocurrent of 5.19 mA/cm^2^ (at 1.23 V_RHE_) for PEC hydrogen production compared to that of the bare BiVO_4_ (4.88 mA/cm^2^).

## 2. Materials and Methods

### 2.1. Preparation of Nanoporous BiVO_4_ Films

Nanoporous BiVO_4_ films were prepared via a typical electrodeposition process, following a previously reported method [11]. Fluorine-doped tin oxide (FTO) glasses (TEC-8, Pilkington) were cleaned using ethyl alcohol in an ultrasonic bath for 30 min and subsequently treated with UV/O_3_ (Yuil Ultraviolet System Inc.) for 20 min to remove surface contaminants. For the electrodeposition of BiOI films, a 0.04 M aqueous solution of Bi(NO_3_)_3_ was prepared by dissolving Bi(NO_3_)_3_·5H_2_O (Daejung) in a 0.4 M KI (Daejung) aqueous solution with pH adjusted to 1.7 using HNO_3_ (Daejung). A 20 mL ethanolic solution of 0.23 M p-benzoquinone (Daejung) was added to this solution and vigorously stirred to ensure complete mixing. The electrodeposition was carried out using a potentiostat (Multi Autolab M204, Metrohm) with a three-electrode configuration consisting of the cleaned FTO glasses as the working electrode and a Pt mesh and an Ag/AgCl electrode as the counter and reference electrodes, respectively, in the prepared Bi(NO_3_)_3_ solution. Electrodeposition was performed at –0.1 V vs. Ag/AgCl at room temperature (RT) for 4 min, followed by washing the surface of the electrodeposited BiOI films with deionized (DI) water and drying at RT. Subsequently, a 0.2 M VO(acac)_2_ (Sigma-Aldrich) solution in dimethyl sulfoxide (DMSO, Kanto) was dropped onto the BiOI films with an amount of ~100 μL/cm^2^, and the resulting films were annealed at 450 °C for 2 h in air. To remove excess V_2_O_5_ on the BiVO_4_ surface, the annealed BiVO_4_ films were stirred in a 1 M NaOH (Daejung) aqueous solution for 30 min, followed by washing with DI water and drying at RT.

### 2.2. Deposition of PbS QDs on the Surface of BiVO_4_ Films

The deposition of PbS QDs onto the BiVO_4_ films was performed using a SILAR method, which was previously reported [17,26]. The FTO/BiVO_4_ electrodes were immersed in a 0.02 M methyl alcohol solution of Pb(NO_3_)_2_ (Sigma-Aldrich) for 90 s, followed by immersion in a solution of 0.02 M Na_2_S (Sigma-Aldrich) in methanol/DI water (1:1, *v*/*v*) for 90 s. After each dipping, the electrodes were thoroughly washed with methyl alcohol, and the SILAR cycle was repeated 3–7 times. To apply ZnS overlayers on the BiVO_4_/PbS QDs films, the electrodes were alternatively immersed in a 0.06 M ethyl alcohol solution of Zn(NO_3_)_2_·6H_2_O (Sigma-Aldrich) and a 0.06 M solution of Na_2_S (Sigma-Aldrich) in methanol/DI water (1:1, *v*/*v*) for 50 s each. After each dipping, the electrodes were thoroughly washed with methyl alcohol, and the SILAR cycle was conducted three times.

### 2.3. Characterization

The surface morphology and structure of the electrodes were characterized using various analytical techniques. A field-emission scanning electron microscope (FE-SEM, S-4700, Hitachi) and high-resolution transmission electron microscopy (HR-TEM; JEM-2010, JEOL) were utilized to examine the surface morphology and structure. Elemental mapping was conducted using a SEM (CX-200, COXEM) equipped with an energy-dispersive X-ray spectroscopy (EDX) detector. X-ray diffraction (XRD) analyses were performed using an X-ray diffractometer (SmartLab 9 kW system, Rigaku). The chemical and electronic states of the electrodes were investigated using X-ray photoelectron spectroscopy (XPS, K-alpha+, Thermo Fisher). The UV-vis absorption spectra of the BiVO_4_/PbS QDs films were obtained with UV-vis spectroscopy (OPTIZEN 2120 UV, KLAB). Steady-state photoluminescence (PL) spectra were recorded using a fluorescence spectrophotometer (FlouTime 300, PicoQuant). The PEC performances were measured using a potentiostat (Multi Autolab M204, Metrohm) with a three-electrode configuration consisting of a BiVO_4_/PbS QDs photoanode and a Pt mesh and SCE electrode as the counter and reference electrodes, respectively, in a quartz reactor. The electrolyte consisted of 0.5 M KH_2_PO_4_ and 1.0 M Na_2_SO_3_ (pH ~7) in DI water. Photocurrent density–voltage (*J-V*) curves were obtained under illumination from a solar simulator (PEC-L01, Peccell) equipped with a 150 W Xe lamp and an AM 1.5G filter. The scan rate was 20 mV/s, and the light intensity of the solar simulator was adjusted to one sun (100 mW/cm^2^) using a NREL-certified Si reference solar cell. Electrochemical impedance spectroscopy (EIS) data were obtained using a frequency response detector in the potentiostat, applying a sinusoidal perturbation of ±10 mV with the frequency varying from 10^−1^ Hz to 10^5^ Hz.

## 3. Results and Discussion

**Figure 1:**
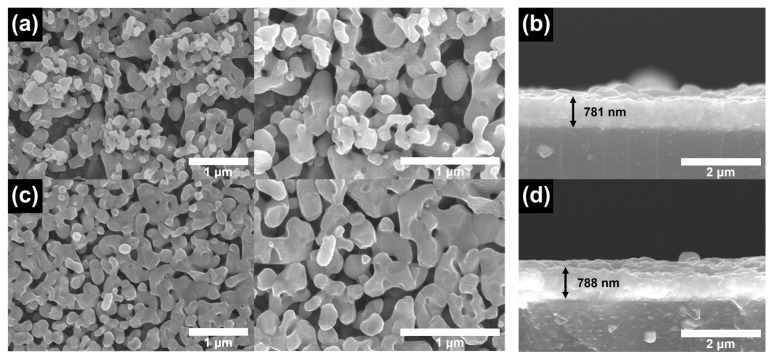
Top and cross-sectional SEM images for (**a**,**b**) bare BiVO_4_ and (**c**,**d**) BiVO_4_/PbS QDs films on FTO glasses.

**Figure 2:**
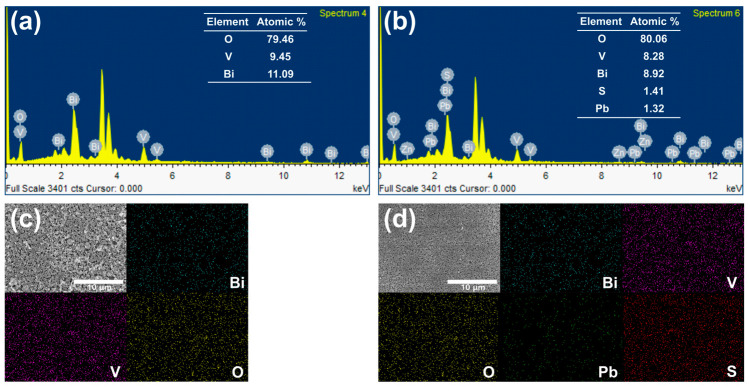
EDX spectra and mapping for each element (O, V, Bi, Pb, and S) of the surface of (**a**,**c**) bare BiVO_4_ and (**b**,**d**) BiVO_4_/PbS QDs films on FTO glasses.

Figure 1a–d present a comparison of the structures and morphologies of bare BiVO_4_ and BiVO_4_/PbS QDs films, prepared via five SILAR cycles and deposited on FTO glasses. The bare BiVO_4_ film exhibits a nanoporous and wormlike structure with main diameters of approximately 200–300 nm, which are interconnected (Figure 1a). After the deposition of PbS QDs, the overall surface structure remains similar to that of the bare sample, but the pore size is slightly reduced and the main diameter marginally increased (Figure 1c). The thickness of both films is nearly the same (Figure 1b,d), indicating that PbS QDs were homogeneously coated on the surface of the nanoporous BiVO_4_. Figure 2 shows the EDX spectra and mapping images for the surfaces of both samples. The atomic ratio between Bi and V is nearly 1:1 for both samples, as indicated by the EDX spectra. Additionally, the atomic ratio between Pb and S is almost 1:1 for the QDs. The mapping images confirm that the PbS QDs were uniformly deposited on the surface of the BiVO_4_ film.

**Figure 3:**
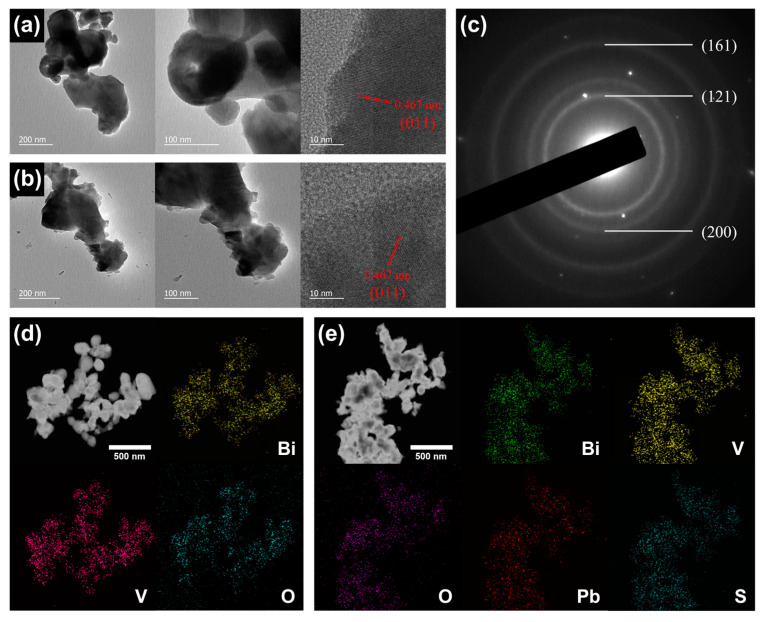
HR-TEM images of (**a**) bare BiVO_4_ and (**b**) BiVO_4_/PbS QDs. (**c**) SAED pattern of bare BiVO_4_. EDX mapping for each element (Bi, V, O, Pb, and S) of (**d**) bare BiVO_4_ and (**e**) BiVO_4_/PbS QDs.

Figure 3a,b present the HR-TEM images of fragments from the bare BiVO_4_ and the BiVO_4_/PbS QDs (prepared via five SILAR cycles) films, respectively. The HR-TEM images for both samples clearly show the (011) lattice plane (fringe spacing ~0.467 nm) of monoclinic BiVO_4_ [13]. Figure 3c also shows the selected area electron diffraction (SAED) pattern of monoclinic BiVO_4_ (JCPDS #14-0688). The EDX mapping in Figure 3d,e confirms the successful synthesis of BiVO_4_ and homogeneous coating of PbS QDs on the surface of nanoporous BiVO_4_. The EDX spectra of each element also indicate the presence of BiVO_4_ and PbS QDs (Figure S1, Supplementary Materials).

**Figure 4:**
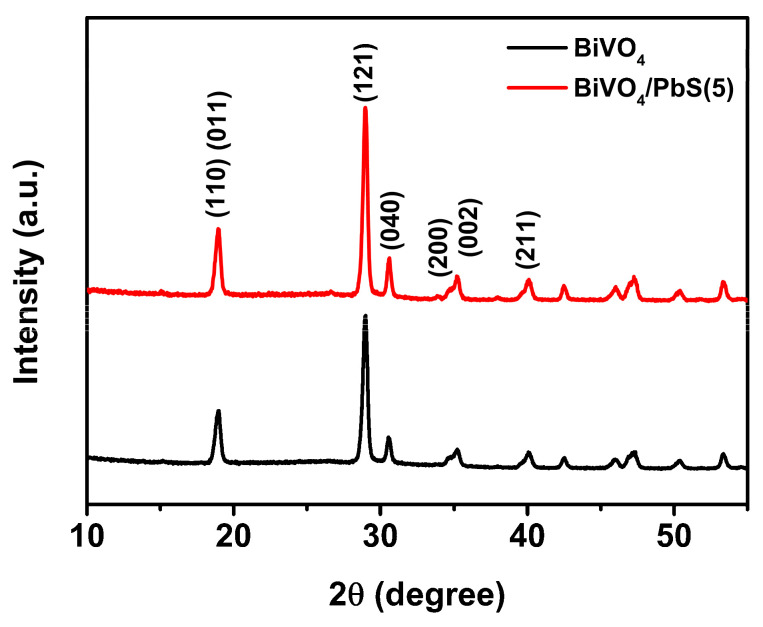
XRD spectra of bare BiVO_4_ and BiVO_4_/PbS QDs films on FTO glasses.

To further characterize the crystal structures of the prepared BiVO_4_/PbS QDs films, XRD spectra were obtained and are presented in Figure 4. Both spectra of the bare BiVO_4_ and the BiVO_4_/PbS QDs films exhibit crystallized monoclinic BiVO_4_ (JCPDS #14-0688), which is consistent with the SAED pattern presented above. In addition, the peak positions of the BiVO_4_/PbS QDs were almost identical to those of the bare BiVO_4_, implying that the PbS QDs were only physically adsorbed on the BiVO_4_ surface and did not affect the crystal structure of BiVO_4_. The XRD peaks corresponding to PbS were not detected due to its poor crystallinity compared to that of BiVO_4_ [29,30].

**Figure 5:**
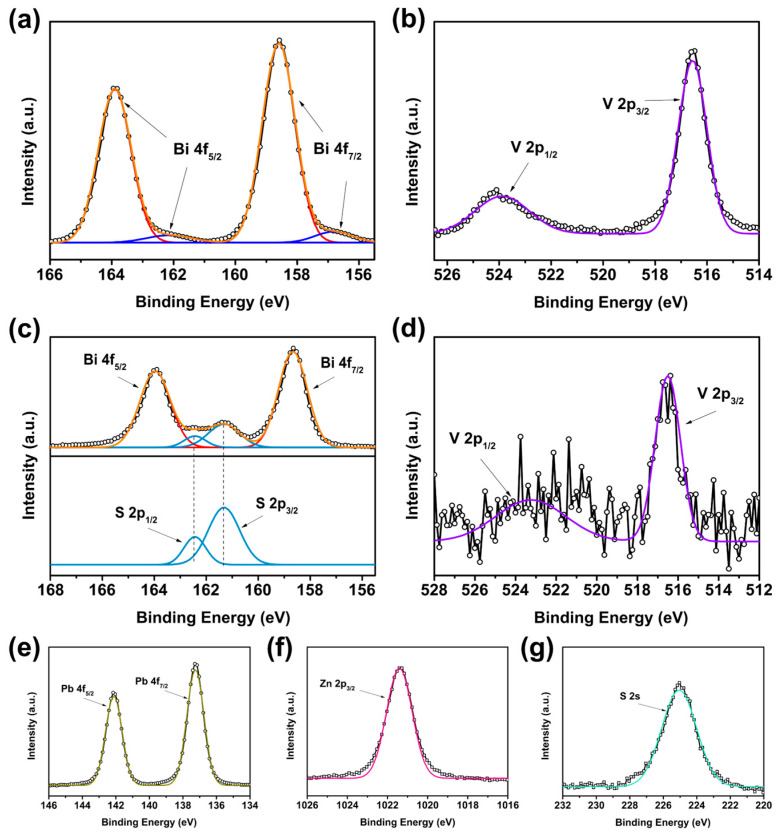
High-resolution XPS spectra of (**a**) Bi 4f and (**b**) V 2p for bare BiVO_4_ film. (**c**) Bi 4f and S 2p, (**d**) V 2p, (**e**) Pb 4f, (**f**) Zn 2p_3/2_, and (**g**) S 2s for BiVO_4_/PbS QDs/ZnS film.

The chemical states of the BiVO_4_/PbS QDs/ZnS films were investigated by XPS analysis. The spectra of the bare BiVO_4_ and BiVO_4_/PbS QDs (prepared via five SILAR cycles)/ZnS films over a wide scan range are shown in Figure S2 in the Supplementary Materials. Both spectra demonstrate the presence of Bi, V, O, and C. In addition, the BiVO_4_/PbS QDs/ZnS film displays extra peaks that correspond to Pb, S, and Zn. Figure 5 displays the high-resolution XPS spectra. In the case of the bare sample, the Bi 4f_7/2_ and 4f_5/2_ peaks exhibit BEs of 158.6 and 163.9 eV, respectively (Figure 5a), indicating the presence of Bi^3+^ in the monoclinic phase of BiVO_4_ [31,32,33]. The minor peaks observed at 156.8 (Bi 4f_7/2_) and 162.1 eV (4f_5/2_) were attributed to the metal species Bi^0^ [33,34,35]. Moreover, the V 2p_3/2_ and 2p_1/2_ peaks have BEs of 516.6 and 523.9 eV (Figure 5b), respectively, which are typical of V^5+^ in BiVO_4_ [31,32,33,34,35,36]. No other significant peaks were observed. Based on the XPS data and the XRD results presented earlier, it can be inferred that most of the Bi and V species existed in the form of the monoclinic phase of BiVO_4_.

The Bi 4f BEs of the BiVO_4_/PbS QDs/ZnS film are similar to those of the bare sample, but the S 2p peaks are overlapped between the Bi 4f peaks (Figure 5c), indicating the presence of PbS QDs. The S 2p_3/2_ and 2p_1/2_ BEs are 161.3 and 162.4 eV, respectively, corresponding to the Pb-S bond [37,38]. The weak V 2p peaks were difficult to analyze accurately due to the presence of PbS QDs/ZnS layers on the BiVO_4_ surface. The Pb 4f_7/2_ and 4f_5/2_ BEs are characteristic of the Pb-S bond, indicating the presence of PbS QDs on the photoanode surface. The Zn 2p_3/2_ peak at 1021.4 eV and the S 2s peak at 225.0 eV correspond to the Zn-S bond of the ZnS overlayer and the Pb-S bond of the PbS QDs, respectively [39,40,41,42].

**Figure 6:**
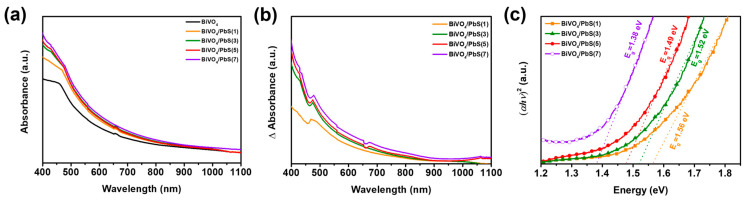
(**a**) Absorption spectra of bare BiVO_4_ and BiVO_4_/PbS(n) QDs films (n: the number of PbS SILAR cycles). (**b**) Absorbance difference between bare BiVO_4_ and BiVO_4_/PbS(n) QDs films. (**c**) Extrapolated plots of (*αhν*)^2^ vs. *hν* achieved from the absorption spectra and utilized to obtain the band -gap of PbS QDs.

To examine the effect of PbS QD coating on the optical properties of the BiVO_4_ film, the absorption spectra were measured and are presented in Figure 6a. The absorbance of the films was enhanced gradually with the increase in the number of PbS SILAR cycles compared to the bare BiVO_4_ film due to the additional light absorption by the deposited PbS QDs. To investigate the absorption property of only the PbS QDs, the difference in absorbance between the bare BiVO_4_ and PbS QD films was compared as a function of the number of PbS SILAR cycles (Figure 6b). The optical band-gap energy (*E*_g_) of the PbS QDs was determined by extrapolating the linear part of (*αhν*)^2^ vs. *hν* plot, where *α* is the absorption coefficient and *hν* is the photon energy (Figure 6c) [17,43]. The *E*_g_ of the PbS QDs decreased gradually as the number of SILAR cycles increased. This means that the size of PbS QDs enlarged gradually as the number of SILAR cycles increased, leading to the reduced *E*_g_ due to the quantum confinement effect [44,45,46]. The *E*_g_ of the PbS QDs (1.38~1.56 eV) was much smaller than that of the BiVO_4_ (~2.4 eV) [12,13,14], allowing the photoelectrodes to utilize the full range of visible light. Additionally, the steady-state PL spectrum of the BiVO_4_ film was not significantly affected by the deposited PbS QDs (Figure S3, Supplementary Materials). The spectral peak position (~545 nm) and the PL intensity are nearly the same between the bare BiVO_4_ and the BiVO_4_/PbS QD films, indicating that the optical property of BiVO_4_ was not influenced by the deposition of PbS QDs.

**Figure 7:**
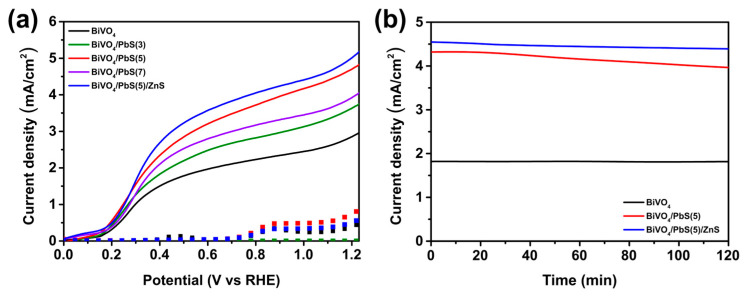
(**a**) J-V curves and (**b**) chronoamperometric curves (at 1.23 V_RHE_) of bare BiVO_4_, BiVO_4_/PbS(n) QDs, and BiVO_4_/PbS(5) QDs/ZnS photoanodes (n: the number of PbS SILAR cycles).

The BiVO_4_/PbS QDs/ZnS films were utilized as the photoanode for PEC hydrogen production and tested under simulated one-sun illumination. The electrolyte used was Ar-purged 0.5 M KH_2_PO_4_ and 1.0 M Na_2_SO_3_ aqueous solution with pH ~7, acting as a hole scavenger to prevent severe photo-corrosion [26]. The J-V curves of each photoanode for PEC hydrogen production according to the number of PbS SILAR cycles are shown in Figure 7a, and the obtained photocurrent densities are summarized in Table 1. It was confirmed that the PbS QDs-sensitized BiVO_4_ photoanodes exhibited improved photocurrent compared to the bare sample, due to the enhanced light-harvesting capability arising from the narrow band-gap of PbS QDs. In particular, the photocurrent was optimized when the SILAR cycles were repeated five times. While the bare BiVO_4_ photoanode exhibited a photocurrent of 2.92 mA/cm^2^ at 1.23 V_RHE_, the BiVO_4_/PbS(5) photoanode exhibited a photocurrent of 4.88 mA/cm^2^. In general, as the number of SILAR cycles is increased, the size of QDs is enlarged, resulting in a smaller band-gap of the QDs [26,47]. Although the absorption range can be extended when the band-gap of the PbS QDs is reduced, the injection efficiency of the photoelectrons from the PbS QDs to the conduction band (CB) of BiVO_4_ can be decreased if the CB of the PbS QDs becomes lower than that of BiVO_4_. This phenomenon has been reported in previous studies with other narrow band-gap QDs such as Cu-In-Se [48]. As the size of these QDs increases, their CB becomes lower than that of the host semiconductor (such as TiO_2_) and results in the poor injection efficiency of photoelectrons in the PEC cells. A similar phenomenon can be expected with the PbS QDs used in this study if their size increases beyond a certain level. Because of this trade-off, the PEC performance of BiVO_4_/PbS QDs/ZnS photoanodes was optimized in the condition of five SILAR cycles, while a higher SILAR cycle (seven cycles) decreased the performance.

Introduction of a passivation layer has been shown to effectively enhance the PEC performance of QD-based electrodes [27]. The most common approach for passivating QD-based electrodes is to deposit ZnS overlayers using the SILAR method [27,28]. As depicted in Figure 7a, the BiVO_4_/PbS(5) photoanode coated with ZnS overlayers (BiVO_4_/PbS(5)/ZnS) exhibited a further enhanced photocurrent of 5.19 mA/cm^2^ compared to the one without overlayers. According to the literature, this enhancement can be attributed to suppressed nonradiative carrier recombination and interfacial electron recombination at the photoanode surface by the ZnS passivation layer [17,27,49].

To assess the photostability of each photoanode, a chronoamperometry test was performed at 1.23 V_RHE_ for 2 h (Figure 7b). As shown in Table 1, the photocurrent density of the bare BiVO_4_ photoanode remained almost unchanged within 2 h of one-sun illumination, owing to the exceptional photostability of BiVO_4_ (the retention rate was ~99.83%). After sensitizing with PbS QDs (via five SILAR cycles), the retention rate of photocurrent density slightly decreased to 91.24%, which can be attributed to the relatively poor photostability of the metal chalcogenide [26,49]. However, after the ZnS overlayer was coated, the retention rate of photocurrent density improved again to 96.20%. This suggests that the ZnS overlayers prevent the photocorrosion of QDs and carrier recombination at the QD surface. To further enhance the photostability, other overlayers such as lead halide ligands and dinickel phosphide (Ni_2_P), which were suggested in the previous literature [50], can be explored in further research. The theoretical hydrogen production was also calculated based on these chronoamperometric curves, as shown in Figure S4 in the Supplementary Materials. The superior photostability and high photocurrent density of BiVO_4_/PbS QDs/ZnS films make them a promising material for highly efficient and reliable PEC hydrogen production. As mentioned previously, this is the first time that narrow band-gap QDs were applied to sensitize a BiVO_4_ photoelectrode, and the performances recorded in this study are comparable to the recently reported excellent performances of BiVO_4_ photoelectrodes (Table S1 in the Supplementary Materials). While several previous studies have reported higher photocurrent values, they employed cocatalysts such as NiFeO_x_, FeOOH, and NiOOH to enhance performance. Further work is needed to identify proper cocatalysts for BiVO_4_/PbS QDs photoelectrodes for even greater enhancements in performance.

**Figure 8:**
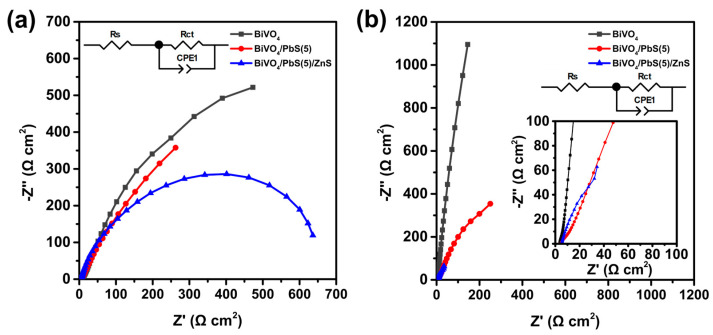
Nyquist plots of bare BiVO_4_, BiVO_4_/PbS(5), and BiVO_4_/PbS(5)/ZnS photoanodes (**a**) in the dark and (**b**) under 1-sun irradiation at 1.23 V_RHE_. The insets present the equivalent circuit model for the analysis of EIS data.

In order to gain a deeper understanding of the effects of PbS QDs and ZnS overlayers on the performance of the PEC system, EIS analysis was conducted under dark conditions and one-sun irradiation (Figure 8). The impedance spectra were analyzed using Z-view software based on an equivalent circuit model, as shown in the insets. This model includes a solution resistance (*R*_S_) and a RC circuit consisting of a charge transfer resistance (*R*_ct_) and a constant phase element (CPE1) related to the charge transfer properties at the interface between the photoanode and electrolyte [49,51]. The *R*_S_ was similar for all samples, but the *R*_ct_ of the BiVO_4_/PbS(5) photoanode was smaller than that of the bare BiVO_4_ under both conditions (Table 1). This suggests that the poor hole transfer kinetics at the BiVO_4_ surface were improved by the deposition of PbS QDs.

Furthermore, the BiVO_4_/PbS(5)/ZnS photoanode exhibited significantly reduced *R*_ct_ values under both dark and illuminated conditions. This indicates that the surface defect sites on the QDs were effectively passivated by the ZnS overlayer, resulting in a reduction in electron–hole recombination and an enhancement of surface charge transfer [49,51]. Thus, it can be concluded that the improved PEC performance of the BiVO_4_ photoanode was attributed not only to the narrow band-gap of PbS, but also to the improved hole transfer properties between the photoanode and electrolyte. Additionally, the passivation by ZnS overlayers was highly effective in reducing electron–hole recombination at the QD surfaces, leading to further enhancement of the PEC performance.

## 4. Conclusions

This study aimed to investigate the effects of PbS QD sensitization on the PEC performance of BiVO_4_ photoanodes. The nanoporous BiVO_4_ films were prepared through electrodeposition, followed by PbS QD sensitization via a SILAR method, which formed a p-n heterojunction. This is the first time that narrow band-gap QDs have been applied to sensitize a BiVO_4_ photoelectrode. The resulting BiVO_4_/PbS QDs photoanode exhibited a significantly increased photocurrent of 4.88 mA/cm^2^ (at 1.23 V_RHE_) for PEC hydrogen production owing to the improved light-harvesting capability from the narrow band-gap of PbS and the enhanced charge transfer properties. Furthermore, when a ZnS overlayer was applied to reduce electron–hole recombination at the QD surface, the photocurrent was further improved to 5.19 mA/cm^2^. These findings provide valuable insights for the development of electrode materials for highly efficient PEC hydrogen production.

## Figures and Tables

**Table 1:** Summary of J-V, chronoamperometric properties, and EIS parameters for bare BiVO_4_, BiVO_4_/PbS(5), and BiVO_4_/PbS(5)/ZnS photoanodes. All data were achieved at 1.23 V_RHE_.

Photoanode	Photocurrent Density (mA/cm^2^)	Photocurrent Density Retention after 2 h (%)	*R*_S_ (Ω cm^2^) (Dark)	*R*_ct_ (Ω cm^2^) (Dark)	*R*_S_ (Ω cm^2^) (Light)	*R*_ct_ (Ω cm^2^) (Light)
BiVO_4_	2.92	99.83	5.38	3091	5.07	2541
BiVO_4_/PbS(5)	4.88	91.24	5.15	2562	4.96	2183
BiVO_4_/PbS(5)/ZnS	5.19	96.20	5.14	724	5.05	330

## Data Availability

All data are contained within the article.

## Supplementary Materials

The following are available online at https://www.mdpi.com/article/10.3390/nano13050799/s1, Figure S1: TEM images and relevant EDX spectra of each element with the relative concentration for (a) bare BiVO_4_ and (b) BiVO_4_/PbS QDs, Figure S2: Survey XPS scans of bare BiVO_4_ and BiVO_4_/PbS QDs/ZnS films, Figure S3: Steady-state PL spectra of bare BiVO_4_ and BiVO_4_/PbS(n) QDs films (n: the number of PbS SILAR cycles), Figure S4: Calculated theoretical hydrogen production of bare BiVO_4_, BiVO_4_/PbS(5) QDs, and BiVO_4_/PbS (5) QDs/ZnS photoanodes, based on chronoamperometric curves (at 1.23 V_RHE_), Table S1: Previously reported performances of BiVO_4_ photoelectrodes for PEC hydrogen production. References [11,14,15,52,53,54,55,56] are cited in the supplementary materials.
