# Supplementary material for: PbS Quantum Dots-Decorated BiVO4 Photoanodes for Highly Efficient Photoelectrochemical Hydrogen Production
Source: Nanomaterials (Basel). 2023 Feb 22;13(5):799. doi: 10.3390/nano13050799 (PMC10005482; doi:10.3390/nano13050799)
Supplement: Supplementary file 1 [file nanomaterials-13-00799-s001.zip › nanomaterials-2183648-supplementary.pdf]

nanomaterials

MDPI

Supplementary Materials

PbS Quantum Dots Decorated $BiVO_4$ Photoanodes for Highly Efficient Photoelectrochemical Hydrogen Production

Joo-Won Seo †, Seung-Beom Ha †, In-Cheul Song and Jae-Yup Kim *

Department of Chemical Engineering, Dankook University, Yongin 16890, Republic of Korea

* Correspondence: jykim@dankook.ac.kr

† These authors contributed equally to this work.

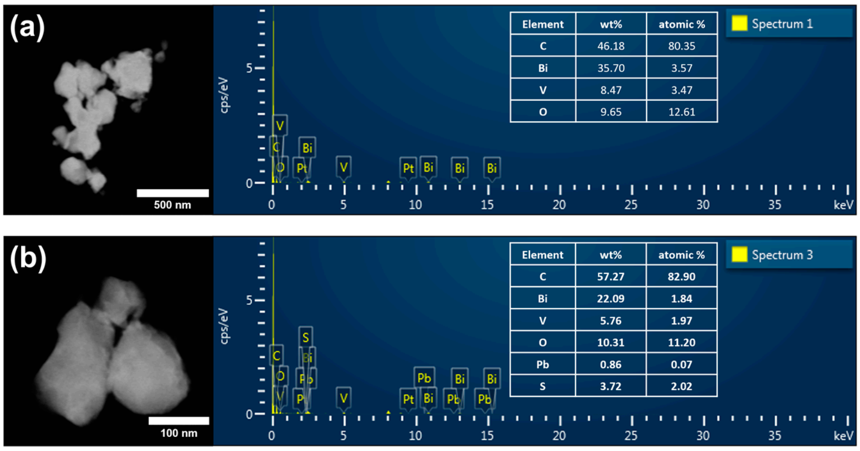

Figure S1. TEM images and relevant EDX spectra of each element with the relative concentration for (a) bare $BiVO_4$ and (b) $BiVO_4$/PbS QDs.

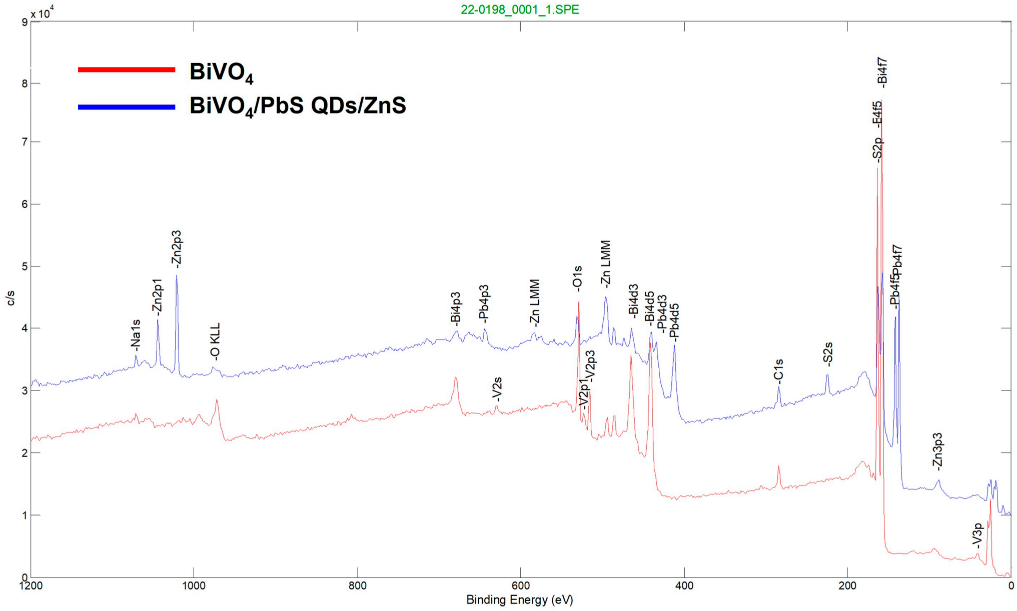

Figure S2. Survey XPS scans of bare $BiVO_4$ and $BiVO_4$/PbS QDs/ZnS films.

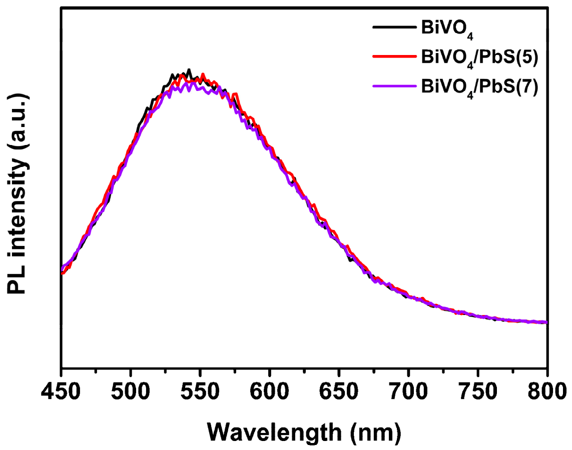

Figure S3. Steady-state PL spectra of bare $BiVO_4$ and $BiVO_4$/PbS(n) QDs films (n : the number of PbS SILAR cycles).

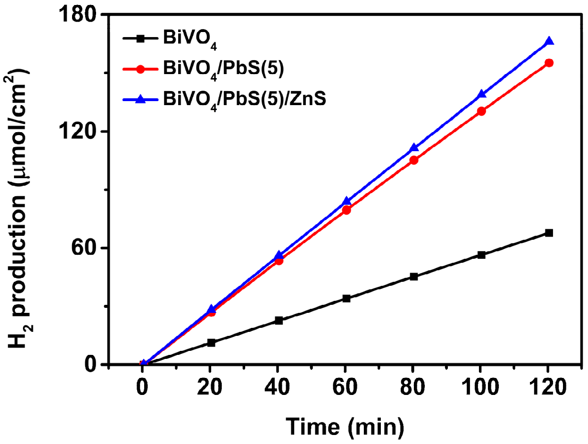

Figure S4. Calculated theoretical hydrogen production of bare $BiVO_4$, $BiVO_4$/PbS(5) QDs, and $BiVO_4$/PbS(5) QDs/ZnS photoanodes, based on chronoamperometric curves (at 1.23 V_{RHE}).

Table S1. Previously reported performances of $BiVO_4$ photoelectrodes for PEC hydrogen production.

Photoelectrode	Electrolyte conditions	Photocurrent density (mA/cm^2)	Retention of photocurrent	Reference
$BiVO_4/NiFeO_x$	1.0 M borate buffer (pH = 9.5)	6.24 (at 1.23 V vs. RHE)	~100 % (after 80 h)	[14]
$TiO_{2-x}/BiVO_4$	0.5 M KPi buffer (pH = 7)	6.12 (at 1.23 V vs. RHE)	~ 83 % (after 100 h)	[15]
Mo-$BiVO_4$/B-$C_3N_4/NiFeO_x$	potassium phosphate buffer with 0.5 M Na_2SO_3 (pH=7)	~6 (at 1.23 V vs. RHE)	92% (after 10 h)	[52]
$BiVO_4$/FeOOH/NiOOH	1.0 M potassium borate with 0.2 M Na_2SO_3 (pH = 9.5)	5.87 (at 1.23 V vs. RHE)	~ 97 % (after 10 h)	[53]
$BiVO_4$/FeOOH	0.5 M borate buffer (pH = 9.5)	4.71 (at 1.23 V vs. RHE)	~ 100 % (after 11 h)	[54]
Ar plasma treated $BiVO_4$	potassium phosphate buffer with 1.0 M Na_2SO_3 (pH=7)	4.32 (at 1.23 V vs. RHE)	~ 100% (after 45h)	[55]
$BiVO_4$/Co(salophen)	0.1 M phosphate buffer (pH=7)	4.27 (at 1.23 V vs. RHE)	~ 100 % (after 3h)	[56]
$BiVO_4$/FeOOH/NiOOH	0.5 M phosphate buffer (pH=7)	4.2 (at 1.23 V vs. RHE)	~ 100 % (after 48h)	[11]
**$BiVO_4$/PbS/ZnS**	**0.5 M KH_2PO_4 with 1.0 M Na_2SO_3 (pH = 7)**	**5.19 (at 1.23 V vs. RHE)**	**~ 97 % (after 2h)**	**This work**
